# miRNAs and lncRNAs as Predictive Biomarkers of Response to FOLFOX Therapy in Colorectal Cancer

**DOI:** 10.3389/fphar.2018.00846

**Published:** 2018-08-06

**Authors:** Kha Wai Hon, Nadiah Abu, Nurul-Syakima Ab Mutalib, Rahman Jamal

**Affiliations:** UKM Medical Molecular Biology Institute (UMBI), Universiti Kebangsaan Malaysia, Kuala Lumpur, Malaysia

**Keywords:** FOLFOX, chemo-resistance, biomarkers, non-coding RNAs, molecular target

## Abstract

Chemotherapy is one of the options for cancer treatment. FOLFOX is one of the widely used chemotherapeutic regimens used to treat primarily colorectal cancer and other cancers as well. However, the emergence of chemo-resistance clones during cancer treatment has become a critical challenge in the clinical setting. It is crucial to identify the potential biomarkers and therapeutics targets which could lead to an improvement in the success rate of the proposed therapies. Since non-coding RNAs have been known to be important players in the cellular system, the interest in their functional roles has intensified. Non-coding RNAs (ncRNAs) as regulators at the post-transcriptional level could be very promising to provide insights in overcoming chemo-resistance to FOLFOX. Hence, this mini review attempts to summarize the potential of ncRNAs correlating with chemo-sensitivity/resistance to FOLFOX.

## Introduction

Colorectal cancer (CRC) is the third most commonly diagnosed cancer and the fourth leading cause of cancer mortality worldwide (Torre et al., 2015). CRC begins with the appearance of benign adenomatous polyps on the inner wall of colon and rectum in large intestine, which progressively develops into advanced adenoma, invasive carcinoma and eventually distant metastases (Flor et al., 2013; Yee et al., 2013; US Preventive Services Task Force et al., 2016; Veettil et al., 2016). Despite the advancement in clinical oncology, multidrug resistance (MDR) remains a major obstacle for treatment of CRC patients especially those at the advanced stage of the disease (Hammond et al., 2016). Several mechanisms have been proposed to modulate MDR in CRC, mainly via limitation of drug transport, dysregulation of cellular processes, alteration of drug sensitivity via epigenetic modifications such as disturbance of miRNA levels and others (Holohan et al., 2013; Panczyk, 2014; Hu et al., 2016; Zhang and Wang, 2017). Over-expression of ATP-dependent transporters on plasma membrane of cancer cells could be responsible for suppressing the influx of drug into cancer cells, and simultaneously increasing the efflux of drug out of the cancers cells to reduce overall drug accumulation (Liu et al., 2010; Wilson et al., 2011; Nies et al., 2015). Dysregulation of cellular processes including apoptosis, drug metabolism, DNA damage repair and regulation of cell cycle checkpoints may also modulate MDR in CRC (Bouwman and Jonkers, 2012; Xu et al., 2012, 2013; Czabotar et al., 2014). Epigenetic modification in cancer cells such as, selective expression of miRNAs, DNA methylation and histone modification have been postulated to alter drug sensitivity in certain cancers including CRC (Brown et al., 2014; Shen et al., 2017; Zhang and Wang, 2017). There are various mechanisms that lead to drug resistance in CRC, but nevertheless, this issue still remains widely unresolved.

One of the CRC chemotherapeutic regimens being widely used today is FOLFOX, which is the combination of folinic acid (FOL), 5-fluorouracil (F) and oxaliplatin (OX) (André et al., 2004). 5-fluorouracil (5-FU) as the main component in FOLFOX, is a type of fluoropyrimidine that incorporates into the DNA molecule to inhibit thymidylate synthase (TS) (Longley et al., 2003). This subsequently hinders the synthesis of pyrimidine thymidine required for DNA replication so that actively dividing cancerous cells will undergo apoptosis due to the thymine-less condition (Noordhuis et al., 2004). 5-FU has been used to treat multiple types of cancer including esophageal cancer, gastric cancer, pancreatic cancer, breast cancer, and cervical cancer (Peters et al., 2000; Ling et al., 2012; Carter et al., 2013; Kim et al., 2016; Lee and Park, 2016). Oxaliplatin (trans-/-diaminocyclohexane oxalatoplatinum; L-OHP) on the other hand, is a platinum-based antineoplastic agent that inhibits DNA replication and transcription by forming cross linkages within the double strands of DNA (Bleiberg, 1998; Woynarowski et al., 2000; Kelland, 2007). The combination of 5-FU and oxaliplatin provide a synergistic effect in anti-proliferative activity, especially among patients with metastatic colorectal cancer (mCRC) (Gustavsson et al., 2015). Folinic acid, also known as leucovorin or calcium folinate, stabilizes the 5-FU-TS complex with better cytotoxicity, and it acts by reducing the side effects of 5-FU with lower dosage required to complete the cycles of treatment (Morgan, 1989; Van Der Wilt et al., 1992). Leucovorin and oxaliplatin also exhibit antitumor properties against metastatic colorectal cancer, esophageal cancer, gastric cancer and hepatocellular carcinoma, and are either used individually or in combination with other chemotherapeutics (Lin et al., 2013b;Skinner et al., 2014; Wu et al., 2014a;Hironaka et al., 2016; Liu et al., 2016b). Currently, FOLFOX is widely administered through injection into the veins to treat mostly stage II and III CRC patients after surgical resection (André et al., 2015). Although FOLFOX is among the preferred chemotherapeutic regimen for CRC patients, the response rate to this systemic treatment is only estimated at around 50%. Studies have reported that almost half of the patients receiving FOLFOX develop chemo-resistance at a later stage of treatment, resulting in high incidence rate of cancer recurrence and metastasis to other organs (De Gramont et al., 2000; André et al., 2004, 2015; Howlader et al., 2016).

Non-coding RNAs (ncRNAs) represent a group of functional RNA molecules originally transcribed from DNA but not translated into proteins (Chen and Xue, 2016). ncRNAs can be classified into two major groups: infrastructural and regulatory ncRNAs (Kaikkonen et al., 2011). Infrastructural ncRNAs such as transfer RNAs (tRNAs), ribosomal RNAs (rRNAs) and small nuclear RNAs (snRNAs) are abundantly expressed in all eukaryotic cells and p2lay housekeeping roles in splicing and translation of mRNAs into proteins (Mattick and Makunin, 2006). Regulatory ncRNAs include microRNA (miRNA), short interfering RNAs (siRNA), piwi-interacting RNAs (piRNA), and long ncRNAs (lncRNAs). They are involved in the epigenetic modification of other RNAs (Fu, 2014). These regulatory ncRNAs regulate gene expression at the transcriptional and post-transcriptional level, via several mechanisms namely heterochromatin formation, histone modification, DNA methylation, and gene silencing (Meister and Tuschl, 2004; Volpe and Martienssen, 2011; Nohata et al., 2013; Matzke and Mosher, 2014). Other classes of RNAs have been discovered in recent decade, such as enhancer RNAs (eRNAs), circular RNAs (circRNAs) and promoter-associated RNAs (PARs), but the limited understanding on these minor classes of RNAs requires more studies to validate their functions in gene regulation (Han et al., 2007; Yan and Ma, 2012; Kim et al., 2015; Salzman, 2016). MicroRNAs (miRNAs) are a class of small, single stranded endogenous ncRNAs with 21–25 nucleotides (Ul Hussain, 2012). MiRNAs bind partially or completely with complementary sequences of the target mRNAs and can silence the mRNA through regulatory mechanisms such as cleavage of the mRNA strand and destabilization of the mRNA through shortening of its poly(A) tail (Bartel, 2009). MiRNAs play important roles in a variety of biological processes, namely cellular development, proliferation, differentiation, metabolism, apoptosis and tumorigenesis (Ul Hussain, 2012). Long non-coding RNAs (lncRNA) constitute a large family of ncRNAs with a length of 200 nucleotides and longer (Geisler and Coller, 2013). LncRNAs mostly interact with DNA, RNA and proteins on the secondary and tertiary structures to form multiple kinds of complexes that could be key regulators in modulating gene expression (Mercer et al., 2009). Aberrant expression of lncRNAs has been discovered in many diseases including cancers (Fang and Fullwood, 2016). Emerging literature has revealed the importance of lncRNAs as oncogenes or tumor suppressors to regulate several key steps in the process of carcinogenesis, such as tumor proliferation, apoptosis, metastasis and chemo-resistance by interfering with target gene expression (Gupta et al., 2010; Yang et al., 2012; Majidinia and Yousefi, 2016; Pan et al., 2016). Concurrently, lncRNA may also serve as therapeutic targets or biomarkers of disease pathogenesis and pathophysiology (Lavorgna et al., 2016). Recent discoveries from transcriptomic and bioinformatics studies have reported increasing number of miRNAs and lncRNAs which modulate epigenetic regulation of cancer chemo-resistance. Emerging evidence has also demonstrated that interactions between miRNAs and lncRNAs with other biomolecules such as proteins are equally important to modulate the molecular mechanism underlying cancer chemo-resistance. This review provides insight into promising miRNAs and lncRNAs as potential biomarkers or therapeutic targets related to FOLFOX-responsiveness in colorectal cancer.

### miRNAs and folfox chemo-resistance

Chen et al. reported that the upregulation of serum miR-19a is significantly associated with FOLFOX-resistance in advanced CRC (Chen et al., 2013b). In this study, serum miR-19a was reported to have a sensitivity of 66.7% and specificity of 63.9% to differentiate FOLFOX-resistant patients from FOLFOX-responsive patients. This implicates the potential of miR-19a as a biomarker in advanced CRC to predict innate resistance before FOLFOX therapy as well as to monitor the acquired resistance of FOLFOX during the treatment (Chen et al., 2013b). MiR-19a is an integral component of the oncomiRs—miR-17-92 family (miR-17, miR-18a, miR-19a, miR-20a, miR-19b-1, and miR-92-1) (Olive et al., 2009; Matsumura et al., 2015). The aberrant expression of this oncogenic cluster has been observed in different cancers, such as myeloma, acute myeloid leukemia, lung cancer, bladder cancer and CRC (Zhang et al., 2012; Collins et al., 2013; Lepore et al., 2013; Lin et al., 2013a;Feng et al., 2014; Wu et al., 2014b;Yamamoto et al., 2015; Liu et al., 2017). MiR-19a was also detected in CRC-derived exosomes and has been suggested as a possible prognostic biomarker for recurrence in CRC patients (Matsumura et al., 2015). Exosomes are microvesicles released by most of the living cells as natural carriers of molecular information such as DNA and RNA (Milane et al., 2015). Furthermore, miR-19a was also associated with other types of drug resistance, including gefitinib resistance in non-small cell lung cancer (Cao et al., 2017) and epirubicin plus paclitaxel in breast cancer (Li et al., 2014). In breast cancer, miR-19a was involved in resistance by regulating the PTEN protein (Li et al., 2014). Meanwhile, in non-small cell lung cancer, miR-19a was reported to be involved in acquired gefitinib resistance via the c-met pathway (Cao et al., 2017). This is interesting, as this shows that the method of resistance to different drugs is dependent on the type of drug. Different drugs may induce different types of resistance, even though the same miRNA is involved.

Another member of the miR-17-92 cluster, is the miR-17-5p, which has been reported to also be significantly upregulated among FOLFOX-resistant CRC patients (Fang et al., 2014). Elevated expression of miR-17-5p was associated with poor prognosis, distant metastases and advanced clinical presentation (Fang et al., 2014). miR-17-5p could serve as a biomarker to predict chemotherapy response in CRC as well as a potential target for the study of CRC tumorigenesis. Additionally, the role of miR-17-5p in relation to drug resistance was also reported in other types of cancer. For instance, miR-17-5p was involved in erlotinib resistance in non-small cell lung cancer cells (Zhang et al., 2017), paclitaxel resistance in lung cancer (Chatterjee et al., 2014) and cisplatin resistance in gastric cancer(Wang and Wang, 2018). In gastric cancer, the resistance mediated by miR-17-5p was achieved by modulating the p21 protein. In ovarian cancer, miR-17-5p was reported to contribute to drug resistance by regulating the AKT pathway through PTEN, and also other EMT players (Fang et al., 2015). Similarly, in colorectal cancer, the PTEN protein was also found to be involved in miR-17-5p-acquired drug resistance (Fang et al., 2014). Interestingly, it was reported that miR-17-5p affected paclitaxel resistance by binding to the 3'UTR of the beclin-1 gene (Chatterjee et al., 2014). The same observation was seen in erlotinib resistance, where miR-17-5p was reported to bind to the EZH1 gene instead (Zhang et al., 2017). From these reports, it can be postulated that PTEN is a major player in drug resistance, and can be used as a targeted therapy. Additionally, miR-17-5p was found to mediate drug resistance by becoming a competitive inhibitor for different types of genes.

Kjersem et al. also identified the upregulation of three other miRNAs (miR-106a, miR-130b, and miR-484) that could emerge as predictive biomarkers of intrinsic resistance among metastatic CRC patients toward FOLFOX (Kjersem et al., 2014). miR-130b was found to be involved in breast cancer resistance to adriamycin via the PI3K/AKT signaling pathway (Miao et al., 2017). miR-484 was also reported to contribute to gemcitabine resistance in breast cancer and sunitinib resistance in renal cell carcinoma (Merhautova et al., 2015; Ye et al., 2015). In breast cancer, miR484 was found to contribute to resistance by targeting the cell-cycle related protein, CDA (Ye et al., 2015). Additionally, a comprehensive analysis conducted via real time-PCR based profiling of 742 different miRNAs using 26 CRC tissues with or without response to first-line capecitabine and oxaliplatin (XELOX)/FOLFOX treatment reported that the overexpression of miR-27b, miR-181b, and miR-625-3p was significantly associated with poor response to XELOX/FOLFOX (Rasmussen et al., 2013). The same study further validated these candidate miRNAs in primary tumor tissues of 94 metastatic CRC patients, which confirmed that high expression of miR-625-3p to be significantly associated with deprived response to XELOX/FOLFOX as first-line treatment. It was further investigated that this miRNA regulated chemoresistance by targeting MAP2K6 of the MAPK pathway (Lyskjær et al., 2016). Zhang et al screened for differentially expressed miRNAs in the serum of 20 responders and 20 non-responders to FOLFOX (Zhang et al., 2014). They reported that 14 miRNAs were differentially expressed, and the findings were further validated in a larger cohort of patients consisting of 93 responders and 80 non-responders. The validation resulted in the further stratification of potential miRNAs down to five miRNAs. The five serum miRNAs (miR-20a, miR-130, miR-145, miR-216, and miR-372) were further statistically tested whether they can be used to differentiate between the responders and non-responders. The AUC using all five miRNAs were 0.841 in the training set (40 CRC patients) and 0.918 (173 CRC patients), whereas when using CEA and CA19-9, the AUC values were 0.689 and 0.746 respectively. This indicates that the panel of five miRNAs was more accurate at determining the responsiveness toward chemotherapy than CEA and CA19-9(Zhang et al., 2014).

Dong et al. discovered the upregulation of miR-429 in both serum and primary tissues from chemo-resistant CRC patients who received 5-FU based adjuvant chemotherapy including FOLFOX (Dong et al., 2016). Overexpression of miR-429 was positively correlated with tumor size, lymph node involvement, distant metastases and TNM staging, resulting in poor prognosis and lower survival rate for CRC patients. Furthermore, miR-429 was also implicated in cisplatin-resistance in epithelial ovarian cancer (Zou et al., 2017). It was further discovered that the method of cisplatin-resistance was achieved by targeting the ZEB1 protein. This pathway of resistance may be similar to FOLFOX resistance, as both oxaliplatin and cisplatin are platinum-based drugs. Furthermore, Takahashi et al. reported that the expression of miR-148a was lower in non-responder CRC than responders (Takahashi et al., 2012). The lower expression of miR-148a was also associated with lower progression-free survival and significantly poorer overall survival (Takahashi et al., 2012). At the molecular level, the downregulation of miR-148a in primary tissues was correlated with the development of high-grade adenoma and poor clinical outcome in stage III CRC patients (Hibino et al., 2015). All these findings suggest that miR-148a could serve as a predictive biomarker for FOLFOX. For drug resistance, miR-148a was reported to be involved in tamoxifen resistance in breast cancer as well (Chen et al., 2017). In breast cancer, the resistance toward tamoxifen by miR-148a was achieved by targeting the ALCAM protein (Chen et al., 2017).

Liu et al. reported that the reduced expression of serum exosomal miR-4772-3p was significantly associated with a higher risk of tumor recurrence in stage II and stage III CRC patients who received FOLFOX therapy (Liu et al., 2016a). However, no study has been conducted in relation to the levels of miR-4772-3p and FOLFOX-responsiveness. In another study which involved a 3-year follow up that focused on the dynamic monitoring of serum miRNA levels (miR-155, miR-200c, and miR-210) with adjuvant FOLFOX therapy plus cetuximab in 15 CRC patients, the researchers suggested that re-elevation of serum miR-155 levels after surgery and chemotherapy may help to predict chemo-resistance (Chen et al., 2013a). MiR-155 is one of the most multi-functional and conserved miRNA ever reported (Yu et al., 2015; Bayraktar and Van Roosbroeck, 2018). In fact, miR-155 is well known to be associated with resistance of treatment in multiple types of cancer such as breast cancer (Yu et al., 2015), lung cancer (Van Roosbroeck et al., 2017), prostate cancer (Li et al., 2017a) cervical cancer (Lei et al., 2012) and renal cell carcinoma(Merhautova et al., 2015). Though the studies reported different mechanisms on how miR-155 regulate resistance, we can still have a basic view on how miR-155 operates and apply it to further understand its role. MiR-155 is one of the major oncogenic miRNAs that is known to be involved in drug resistance and is well-studied. In breast cancer, miR-155 was found to modulate resistance by targeting the FOXO3 pathway, MAPK pathway and EMT-related proteins (Bayraktar and Van Roosbroeck, 2018). Another study reported the prognostic value of miR-320e as a novel biomarker in CRC and has been validated in two different cohorts of patients treated with FOLFOX (Perez-Carbonell et al., 2015). The expression level of miR-320e in primary CRC tissues showed a positive correlation with recurrence, advanced clinical presentation, poor prognosis among stage II and III CRC patients treated with FOLFOX (Perez-Carbonell et al., 2015).

Recently, a study by Kiss et al. discovered that a cohort of CRC patients treated with the combination of bevacizumab and FOLFOX, showed a distinctive profile of tissue miRNAs (Kiss et al., 2017). The study identified 67 differentially expressed miRNAs between the responders and non-responder, where seven of the miRNAs were independently validated (Kiss et al., 2017). From there, four miRNAs (miR-92b-3p, miR-3156-5p, miR-10a-5p, and miR-125a-5p) were significantly associated with the Response Evaluation Criteria In Solid Tumors (RECIST) criteria of responsiveness (Kiss et al., 2017). Moreover, the combination of these four miRNAs had a sensitivity of 82% and specificity of 64% to differentiate between responders and non-responders, thus indicating the potential use of these miRNAs as biomarkers of chemotherapy responsiveness and progression-free survival (Kiss et al., 2017).

### Long non-coding RNAs (lncRNAs) and folfox-resistance

Li et al. presented two different lncRNAs, namely MALAT1 and HOTAIR that contribute to resistance on 5-FU/oxaliplatin-based chemotherapy via similar inhibition of miR-218 (Li et al., 2017b,c). The lncRNA MALAT1, also known as nuclear-enriched transcript 2 (NEAT2), was initially discovered as a promising biomarker for lung cancer metastasis (Gutschner et al., 2013b). Later, discovery of MALAT1 dysregulation was expanded into various cancers to become the key regulator of metastasis and cancer development (Gutschner et al., 2013a;Tripathi et al., 2013). MALAT has also been associated with other types of drug resistance. For instance, MALAT was shown to be involved in cisplatin resistance in NSCLC (Fang et al., 2018), adriamycin resistance in diffuse large-B cell lymphoma (Long et al., 2017) and temozolomide resistance in glioblastoma (Lu et al., 2017). Upregulation of MALAT1 in primary CRC tissue was highly associated with a poor survival rate and a weak response to FOLFOX in advanced CRC patients (Li et al., 2017b). The same study demonstrated that the overexpression of MALAT1 in oxaliplatin-resistant CRC cells modulate chemo-resistance via suppression of E-cadherin expression and enhancement of epithelial-mesenchymal transition (EMT) but the underlying signaling pathways have not been fully elucidated (Wang and Zhou, 2013). Correspondingly, HOTAIR overexpression in primary CRC tissue was also demonstrated to inhibit miR-218 expression in CRC, resulting in poor response to 5FU- based adjuvant chemotherapy (Li et al., 2017c). Similarly, HOTAIR was also reported to be involved in other types of drug resistance in different cancers. It was reported that HOTAIR was associated with cisplatin resistance in lung adenocarcinoma (Liu et al., 2013), crizotinib resistance in NSCLC (Yang et al., 2018) and imatinib resistance in chronic myeloid leukemia (Wang et al., 2017). In gastric cancer, HOTAIR was involved in cisplatin resistance via inhibition of the PI3K/AKT pathway and Wnt/B-catenin pathways. Both of these pathways are hallmark pathways that are involved in colorectal cancer pathogenesis. It can be assumed that FOLFOX resistance was also achieved by the same mechanism. Collectively, Table 1 summarizes all the mentioned ncRNAs that show correlation with chemo-resistance to FOLFOX.

**Table 1:** Summary of ncRNAs associated with resistance to FOLFOX.

**Name**	**Source**	**Cancer model**	**Expression**	**Effect on FOLFOX-resistance**	**Possible significance**	**Reference**
**miRNAs**						
miR-19a	Serum	CRC	Upregulation	Enhancement	To predict innate resistance to FOLFOX	Chen et al., 2013b
miR-4772-3p	Serum exosomes	CRC	Downregulation	Enhancement	High risk of tumor recurrence	Liu et al., 2016a
miR-17-5p	Tissue	CRC	Upregulation	Enhancement	Prognostic factor for overall survival	Fang et al., 2014
	Cell lines		Overexpression	Enhancement	–	
miR-106a miR-130b miR-484	Plasma	CRC	Upregulation	Enhancement	To predict innate resistance to FOLFOX	Kjersem et al., 2014
miR-27b miR-181b miR-625-3p	Tissue	CRC	Upregulation	Enhancement	Poor prognosis	Rasmussen et al., 2013
miR-20a miR-130 miR-145 miR-216 miR-372	Serum	CRC	Upregulation	Enhancement	Predictor for chemo-sensitivity	Zhang et al., 2014
miR-429	Tissue Serum	CRC	Upregulation	Enhancement	Poor prognosis	Dong et al., 2016
miR-425-5p	Cell line	CRC	Upregulation	Enhancement	-	Zhang et al., 2016
miR-148a	Tissue	CRC	Downregulation	Enhancement	Poor prognosis	Takahashi et al., 2012
miR-155	Serum	CRC	Upregulation	Enhancement	Predictor for chemo-resistance	Chen et al., 2013a
miR-320e	Tissue	CRC	Upregulation	Enhancement	Poor prognosis	Perez-Carbonell et al., 2015
miR-139-5p	Tissue Serum	CRC	Upregulation	Enhancement	To predict cancer recurrence and distant metastasis	Miyoshi et al., 2017
miR-92b-3p, miR-3156-5p, miR-10a-5p, and miR-125a-5p	Tissue	CRC	Upregulation	Enhancement	Predictor for chemo-resistance	Kiss et al., 2017
**lncRNAs**						
MALAT1	Tissue	CRC	Upregulation	Enhancement	Downregulate miR-218	Li et al., 2017b
	Cell line				–	
HOTAIR	Tissue	CRC	Upregulation	Enhancement	Downregulate miR-218	Li et al., 2017c

## Conclusion, challenges and future direction

**Figure 1:**
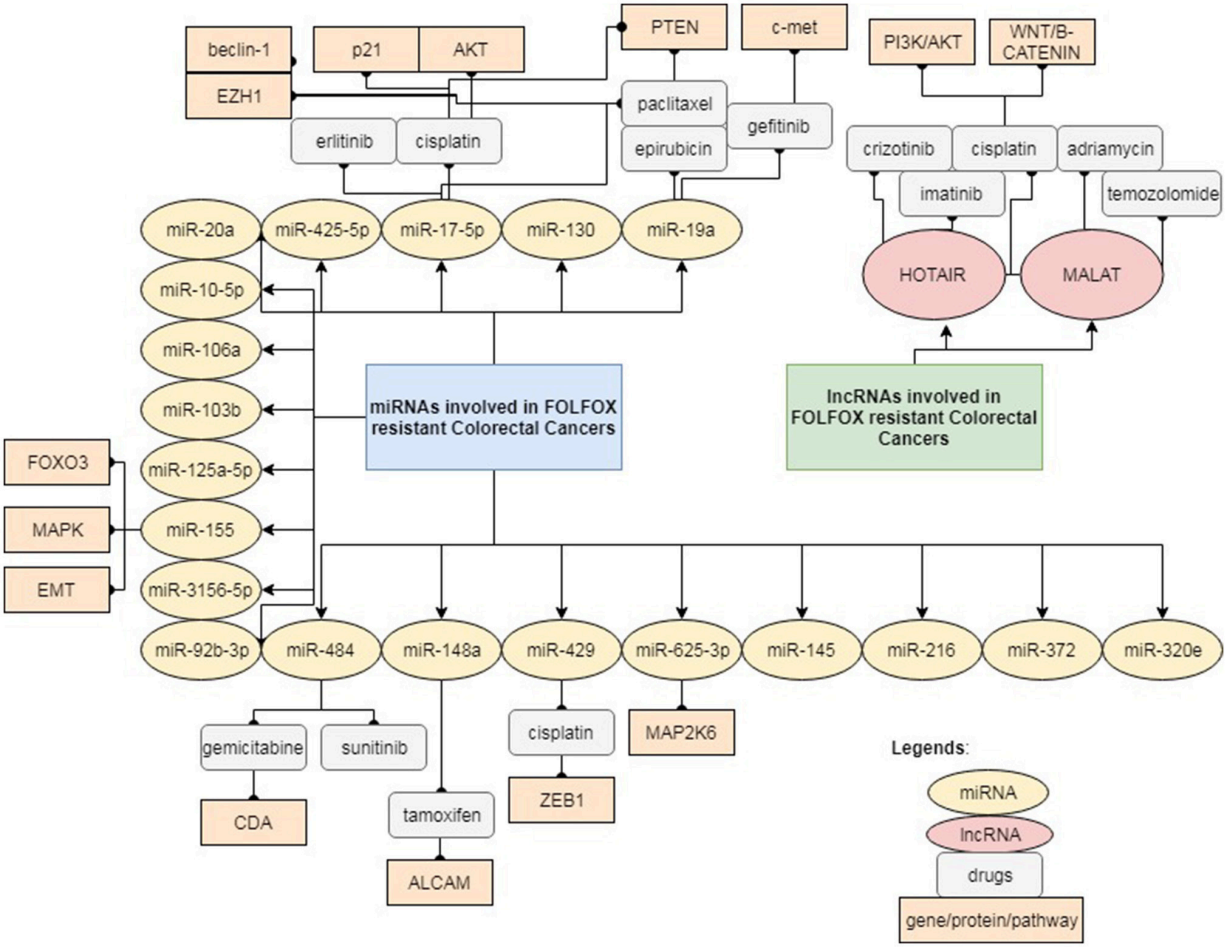
Reported ncRNAs and the targeted genes/proteins that may affect FOLFOX responsiveness.

This mini-review highlights the increasing evidence and a fresh update that will help to widen our knowledge on the potential role of ncRNAs, primarily miRNAs and lncRNAs underlying FOLFOX chemo-resistance. Most of the ncRNAs reported are not only involved in FOLFOX resistance, but in resistance to other drugs as well. This reflects that the mechanisms of drug resistance are rather complex and different pathways may crosstalk with each other as illustrated in Figure 1. Drug resistance may also occur in a centralized manner, regardless of the type of cancer or drug being administered. Nevertheless, there are also instances, where a certain ncRNA may modulate different pathways of resistance depending on the type of drugs. Most of the studies mentioned above were performed on analysis of clinical tissues and serum/plasma samples from retrospective patient cohorts without sufficient validation and in-depth functional analysis. Future research is necessary to validate these findings in multi-centered cohort studies as well as to elucidate the underlying signaling pathway via *in vitro* and *in vivo* functional studies. The bioinformatics analysis in studies related to FOLFOX-resistance is still insufficient to provide solid foundation for the translation of miRNAs and lncRNAs as powerful predictor of FOLFOX-resistance in the clinical setting. More intensive and comprehensive statistical analysis is essential to validate the specificity and sensitivity of each individual miRNA/lncRNA as a biomarker (Liu et al., 2012). Ultimately, all these findings may contribute toward the development of next-generation diagnostic panel comprising of miRNAs and lncRNAs, which a more powerful diagnostic tool to predict patient response toward FOLFOX. However, it is still challenging to accurately identify those clinically promising ncRNAs suitable for early diagnosis, risk assessment, prognosis prediction and drug monitoring in patients treated with FOLFOX. Furthermore, there are still considerable obstacles that limit the clinical application of miRNAs and lncRNAs for diagnostic and prognostic purposes. Lack of standardization in the extraction of ncRNAs from tissues and bodily fluids remains a major challenge, which greatly affects the stability of ncRNAs in specimens and subsequently leads to inconsistency in most findings. Due to the high abundance of ncRNAs in human serum/plasma, the liquid biopsy approach could be an ideal method to develop standardized operative procedures for ncRNAs extraction in the clinical environment (Erbes et al., 2015; Komatsu et al., 2015). Liquid biopsy is now widely accepted as a non-invasive method to retrieve circulating cancer cells or traces of nuclei acids derived from tumor (Karachaliou et al., 2015; Murtaza et al., 2015; Birkenkamp-Demtröder et al., 2016; Jamal-Hanjani et al., 2016). Discovery and characterization of new ncRNAs related to FOLFOX-resistance will benefit the researchers to explore the diverse ncRNAs as potential biomarkers and therapeutic targets to overcome drug resistance.

## Author contributions

KH and NA drafted the manuscript. NA, N-SA, and RJ were responsible for critical feedback and manuscript revision.

### Conflict of interest statement

The authors declare that the research was conducted in the absence of any commercial or financial relationships that could be construed as a potential conflict of interest.

## References

[B1] André T. Boni C. Mounedji-Boudiaf L. Navarro M. Tabernero J. Hickish T.. (2004). Oxaliplatin, fluorouracil, and leucovorin as adjuvant treatment for colon cancer. N. Engl. J. Med. 350, 2343–2351. 10.1056/NEJMoa032709 15175436

[B2] André T. De Gramont A. Vernerey D. Chibaudel B. Bonnetain F. Tijeras-Raballand A.. (2015). Adjuvant fluorouracil, leucovorin, and oxaliplatin in stage ii to iii colon cancer: updated 10-year survival and outcomes according to braf mutation and mismatch repair status of the MOSAIC study. J. Clin. Oncol. 33, 4176–4187. 10.1200/JCO.2015.63.4238 26527776

[B3] Bartel D. P. (2009). MicroRNAs: target recognition and regulatory functions. Cell 136, 215–233. 10.1016/j.cell.2009.01.002 19167326 PMC3794896

[B4] Bayraktar R. Van Roosbroeck K. (2018). miR-155 in cancer drug resistance and as target for miRNA-based therapeutics. Cancer Metastasis Rev. 37, 33–44. 10.1007/s10555-017-9724-7 29282605

[B5] Birkenkamp-Demtröder K. Nordentoft I. Christensen E. Høyer S. Reinert T. Vang S.. (2016). Genomic alterations in liquid biopsies from patients with bladder cancer. Eur. Urol. 70, 75–82. 10.1016/j.eururo.2016.01.007 26803478

[B6] Bleiberg H. (1998). Oxaliplatin (L-OHP): a new reality in colorectal cancer. Br. J. Cancer 77, 1–3. 10.1038/bjc.1998.427 9647611 PMC2149880

[B7] Bouwman P. Jonkers J. (2012). The effects of deregulated DNA damage signalling on cancer chemotherapy response and resistance. Nature Rev. Cancer 12, 587–598. 10.1038/nrc3342 22918414

[B8] Brown R. Curry E. Magnani L. Wilhelm-Benartzi C. S. Borley J. (2014). Poised epigenetic states and acquired drug resistance in cancer. Nature Rev. Cancer 14, 747–753. 10.1038/nrc3819 25253389

[B9] Cao X. Lai S. Hu F. Li G. Wang G. Luo X.. (2017). miR-19a contributes to gefitinib resistance and epithelial mesenchymal transition in non-small cell lung cancer cells by targeting c-Met. Sci. Rep. 7:2939. 10.1038/s41598-017-01153-0 28592790 PMC5462753

[B10] Carter S. K. Sakurai Y. Umezawa H. (2013). New Drugs in Cancer Chemotherapy. Berlin; Heidelberg: Springer.

[B11] Chatterjee A. Chattopadhyay D. Chakrabarti G. (2014). miR-17-5p downregulation contributes to paclitaxel resistance of lung cancer cells through altering beclin1 expression. PLoS ONE 9:e95716. 10.1371/journal.pone.0095716 24755562 PMC3995800

[B12] Chen J. Wang W. Zhang Y. Chen Y. Hu T. (2013a). Predicting distant metastasis and chemoresistance using plasma miRNAs. Med. Oncol. 31:799 10.1007/s12032-013-0799-x 24310813

[B13] Chen J. Xue Y. (2016). Emerging roles of non-coding RNAs in epigenetic regulation. Sci. China Life Sci. 59, 227–235. 10.1007/s11427-016-5010-0 26825947

[B14] Chen M. J. Cheng Y.-M. Chen C.-C. Chen Y.-C. Shen C.-J. (2017). MiR-148a and miR-152 reduce tamoxifen resistance in ER+ breast cancer via downregulating ALCAM. Biochem. Biophys. Res. Commun. 483, 840–846. 10.1016/j.bbrc.2017.01.012 28063929

[B15] Chen Q. Xia H. W. Ge X. J. Zhang Y. C. Tang Q. L. Bi F. (2013b). Serum miR-19a predicts resistance to FOLFOX chemotherapy in advanced colorectal cancer cases. Asian Pac. J. Cancer Prev. 14, 7421–7426. 10.7314/APJCP.2013.14.12.7421 24460313

[B16] Collins A. S. Mccoy C. E. Lloyd A. T. O'farrelly C. Stevenson N. J. (2013). miR-19a: an effective regulator of SOCS3 and enhancer of JAK-STAT signalling. PLoS ONE 8:e69090. 10.1371/journal.pone.0069090 23894411 PMC3718810

[B17] Czabotar P. E. Lessene G. Strasser A. Adams J. M. (2014). Control of apoptosis by the BCL-2 protein family: implications for physiology and therapy. Nature Rev. Mol. Cell Biol. 15, 49. 10.1038/nrm3722 24355989

[B18] De Gramont A. Figer A. Seymour M. Homerin M. Hmissi A. Cassidy J. (2000). Leucovorin and fluorouracil with or without oxaliplatin as first-line treatment in advanced colorectal cancer. J. Clin. Oncol. 18, 2938–2947. 10.1200/JCO.2000.18.16.2938 10944126

[B19] Dong S. J. Cai X. J. Li S. J. (2016). The Clinical significance of MiR-429 as a predictive biomarker in colorectal cancer patients receiving 5-fluorouracil treatment. Med. Sci. Monitor Int. Med. J. Exp. Clin. Res. 22, 3352–3361. 10.12659/MSM.900674 27654003 PMC5036382

[B20] Erbes T. Hirschfeld M. Rücker G. Jaeger M. Boas J. Iborra S. (2015). Feasibility of urinary microRNA detection in breast cancer patients and its potential as an innovative non-invasive biomarker. BMC Cancer 15:193 10.1186/s12885-015-1190-4 25886191 PMC4383066

[B21] Fang L. Li H. Wang L. Hu J. Jin T. Wang J.. (2014). MicroRNA-17-5p promotes chemotherapeutic drug resistance and tumour metastasis of colorectal cancer by repressing PTEN expression. Oncotarget 5, 2974–2987. 10.18632/oncotarget.1614 24912422 PMC4102784

[B22] Fang Y. Fullwood M. J. (2016). Roles, functions, and mechanisms of long non-coding RNAs in cancer. Genomics Proteomics Bioinformatics 14, 42–54. 10.1016/j.gpb.2015.09.006 26883671 PMC4792843

[B23] Fang Y. Xu C. Fu Y. (2015). MicroRNA-17-5p induces drug resistance and invasion of ovarian carcinoma cells by targeting PTEN signaling. J. Biol. Res. 22:12 10.1186/s40709-015-0035-2 PMC4619013 26500892

[B24] Fang Z. Chen W. Yuan Z. Liu X. Jiang H. (2018). LncRNA-MALAT1 contributes to the cisplatin-resistance of lung cancer by upregulating MRP1 and MDR1 via STAT3 activation. Biomed. Pharmacother. 101, 536–542. 10.1016/j.biopha.2018.02.130 29505924

[B25] Feng Y. Liu J. Kang Y. He Y. Liang B. Yang P. (2014). miR-19a acts as an oncogenic microRNA and is up-regulated in bladder cancer. J. Exp. Clin. Cancer Res. 33:67 10.1186/s13046-014-0067-8 25107371 PMC4237814

[B26] Flor N. Ceretti A. P. Mezzanzanica M. Rigamonti P. Peri M. Tresoldi S.. (2013). Impact of contrast-enhanced computed tomography colonography on laparoscopic surgical planning of colorectal cancer. Abdom. Imaging 38, 1024–1032. 10.1007/s00261-013-9996-5 23512572

[B27] Fu X.-D. (2014). Non-coding RNA: a new frontier in regulatory biology. Natl. Sci. Rev. 1, 190–204. 10.1093/nsr/nwu008 25821635 PMC4374487

[B28] Geisler S. Coller J. (2013). RNA in unexpected places: long non-coding RNA functions in diverse cellular contexts. Nat. Rev. Mol. Cell Biol. 14, 699–712. 10.1038/nrm3679 24105322 PMC4852478

[B29] Gupta R. A. Shah N. Wang K. C. Kim J. Horlings H. M. Wong D. J.. (2010). Long non-coding RNA HOTAIR reprograms chromatin state to promote cancer metastasis. Nature 464, 1071–1076. 10.1038/nature08975 20393566 PMC3049919

[B30] Gustavsson B. Carlsson G. Machover D. Petrelli N. Roth A. Schmoll H.-J.. (2015). A review of the evolution of systemic chemotherapy in the management of colorectal cancer. Clin. Colorectal Cancer 14, 1–10. 10.1016/j.clcc.2014.11.002 25579803

[B31] Gutschner T. Hämmerle M. Diederichs S. (2013a). MALAT1 — a paradigm for long noncoding RNA function in cancer. J. Mol. Med. 91, 791–801. 10.1007/s00109-013-1028-y 23529762

[B32] Gutschner T. Hämmerle M. Eissmann M. Hsu J. Kim Y. Hung G.. (2013b). The noncoding RNA MALAT1 is a critical regulator of the metastasis phenotype of lung cancer cells. Cancer Res. 73, 1180–1189. 10.1158/0008-5472.CAN-12-2850 23243023 PMC3589741

[B33] Hammond W. A. Swaika A. Mody K. (2016). Pharmacologic resistance in colorectal cancer: a review. Ther. Adv. Med. Oncol. 8, 57–84. 10.1177/1758834015614530 26753006 PMC4699262

[B34] Han J. Kim D. Morris K. V. (2007). Promoter-associated RNA is required for RNA-directed transcriptional gene silencing in human cells. Proc. Natl. Acad. Sci. U.S.A. 104, 12422–12427. 10.1073/pnas.0701635104 17640892 PMC1924466

[B35] Hibino Y. Sakamoto N. Naito Y. Goto K. Oo H. Z. Sentani K.. (2015). Significance of miR-148a in colorectal neoplasia: downregulation of miR-148a contributes to the carcinogenesis and cell invasion of colorectal cancer. Pathobiology 82, 233–241. 10.1159/000438826 26389729

[B36] Hironaka S. Sugimoto N. Yamaguchi K. Moriwaki T. Komatsu Y. Nishina T.. (2016). S-1 plus leucovorin versus S-1 plus leucovorin and oxaliplatin versus S-1 plus cisplatin in patients with advanced gastric cancer: a randomised, multicentre, open-label, phase 2 trial. Lancet Oncol. 17, 99–108. 10.1016/S1470-2045(15)00410-6 26640036

[B37] Holohan C. Van Schaeybroeck S. Longley D. B. Johnston P. G. (2013). Cancer drug resistance: an evolving paradigm. Nat. Rev. Cancer 13, 714–726. 10.1038/nrc3599 24060863

[B38] Howlader N. Noone A. M. Krapcho M. Miller D. Bishop K. Altekruse S.F (eds). (2016). SEER Cancer Statistics Review,1975–2013. Bethesda, MD: National Cancer Institute.

[B39] Hu T. Li Z. Gao C. Y. Cho C. H. (2016). Mechanisms of drug resistance in colon cancer and its therapeutic strategies. World J. Gastroenterol. 22, 6876–6889. 10.3748/wjg.v22.i30.6876 27570424 PMC4974586

[B40] Jamal-Hanjani M. Wilson G. A. Horswell S. Mitter R. Sakarya O. Constantin T.. (2016). Detection of ubiquitous and heterogeneous mutations in cell-free DNA from patients with early-stage non-small-cell lung cancer. Annals Oncol. 27, 862–867. 10.1093/annonc/mdw037 26823523

[B41] Kaikkonen M. U. Lam M. T. Y. Glass C. K. (2011). Non-coding RNAs as regulators of gene expression and epigenetics. Cardiovasc. Res. 90, 430–440. 10.1093/cvr/cvr097 21558279 PMC3096308

[B42] Karachaliou N. Mayo-De-Las-Casas C. Molina-Vila M. A. Rosell R. (2015). Real-time liquid biopsies become a reality in cancer treatment. Annals Transl. Med. 3:36. 10.3978/j.issn.2305-5839.2015.01.16 25815297 PMC4356857

[B43] Kelland L. (2007). The resurgence of platinum-based cancer chemotherapy. Nature Rev. Cancer 7, 573–584. 10.1038/nrc2167 17625587

[B44] Kim H. S. Kim J. H. Kim J. W. Kim B. C. (2016). Chemotherapy in elderly patients with gastric cancer. J. Cancer 7, 88–94. 10.7150/jca.13248 26722364 PMC4679385

[B45] Kim T.-K. Hemberg M. Gray J. M. (2015). Enhancer RNAs: a class of long noncoding RNAs synthesized at enhancers. Cold Spring Harb. Perspect. Biol. 7:a018622. 10.1101/cshperspect.a018622 25561718 PMC4292161

[B46] Kiss I. Mlčochová J. Součková K. Fabian P. Poprach A. Halamkova J.. (2017). MicroRNAs as outcome predictors in patients with metastatic colorectal cancer treated with bevacizumab in combination with FOLFOX. Oncol. Lett. 14, 743–750. 10.3892/ol.2017.6255 28693229 PMC5494676

[B47] Kjersem J. B. Ikdahl T. Lingjaerde O. C. Guren T. Tveit K. M. Kure E. H. (2014). Plasma microRNAs predicting clinical outcome in metastatic colorectal cancer patients receiving first-line oxaliplatin-based treatment. Mol. Oncol. 8, 59–67. 10.1016/j.molonc.2013.09.001 24119443 PMC5528512

[B48] Komatsu S. Ichikawa D. Miyamae M. Kawaguchi T. Morimura R. Hirajima S.. (2015). Malignant potential in pancreatic neoplasm; new insights provided by circulating miR-223 in plasma. Expert Opin. Biol. Ther. 15, 773–785. 10.1517/14712598.2015.1029914 25819175

[B49] Lavorgna G. Vago R. Sarmini M. Montorsi F. Salonia A. Bellone M. (2016). Long non-coding RNAs as novel therapeutic targets in cancer. Pharmacol. Res. 110, 131–138. 10.1016/j.phrs.2016.05.018 27210721

[B50] Lee H. S. Park S. W. (2016). Systemic chemotherapy in advanced pancreatic cancer. Gut Liver 10, 340–347. 10.5009/gnl15465 27114434 PMC4849685

[B51] Lei C. Wang Y. Huang Y. Yu H. Huang Y. Wu L.. (2012). Up-regulated miR155 Reverses the Epithelial-mesenchymal transition induced by EGF and increases chemo-sensitivity to cisplatin in human caski cervical cancer cells. PLoS ONE 7:e52310. 10.1371/journal.pone.0052310 23284982 PMC3527539

[B52] Lepore I. Dell'aversana C. Pilyugin M. Conte M. Nebbioso A. De Bellis F. (2013). HDAC inhibitors repress BARD1 isoform expression in acute myeloid leukemia cells via activation of miR-19a and/or b. PLoS ONE 8:e83018 10.1371/journal.pone.0083018 24349422 PMC3859623

[B53] Li B. Jin X. Meng H. Hu B. Zhang T. Yu J.. (2017a). Morin promotes prostate cancer cells chemosensitivity to paclitaxel through miR-155/GATA3 axis. Oncotarget 8, 47849–47860. 10.18632/oncotarget.18133 28599307 PMC5564610

[B54] Li P. Zhang X. Wang H. Wang L. Liu T. Du L.. (2017b). MALAT1 is associated with poor response to oxaliplatin-based chemotherapy in colorectal cancer patients and promotes chemoresistance through EZH2. Mol. Cancer Ther. 16, 739–751. 10.1158/1535-7163.MCT-16-0591 28069878

[B55] Li P. Zhang X. Wang L. Du L. Yang Y. Liu T.. (2017c). lncRNA HOTAIR contributes to 5FU resistance through suppressing miR-218 and activating NF-κB/TS signaling in colorectal cancer. Mol. Ther. Nucleic Acids 8, 356–369. 10.1016/j.omtn.2017.07.007 28918035 PMC5537205

[B56] Li Q. Liu M. Ma F. Luo Y. Cai R. Wang L.. (2014). Circulating miR-19a and miR-205 in serum may predict the sensitivity of luminal a subtype of breast cancer patients to neoadjuvant chemotherapy with epirubicin plus paclitaxel. PLoS ONE 9:e104870. 10.1371/journal.pone.0104870 25137071 PMC4138038

[B57] Lin Q. Chen T. Lin Q. Lin G. Lin J. Chen G.. (2013a). Serum miR-19a expression correlates with worse prognosis of patients with non-small cell lung cancer. J. Surg. Oncol. 107, 767–71 10.1002/jso.23312 23609137

[B58] Lin Y. L. Liau J. Y. Yu S. C. Tseng L. H. Lin L. I. Liang J. T.. (2013b). Oxaliplatin-based chemotherapy might provide longer progression-free survival in kras mutant metastatic colorectal cancer. Transl. Oncol. 6, 363–369. 10.1593/tlo.13166 23730417 PMC3660806

[B59] Ling Y. Chen J. Tao M. Chu X. Zhang X. (2012). A pilot study of nimotuzumab combined with cisplatin and 5-FU in patients with advanced esophageal squamous cell carcinoma. J. Thorac. Dis. 4, 58–62. 10.3978/j.issn.2072-1439.2011.08.02 22295168 PMC3256539

[B60] Liu A. M. Yao T.-J. Wang W. Wong K.-F. Lee N. P. Fan S. T.. (2012). Circulating miR-15b and miR-130b in serum as potential markers for detecting hepatocellular carcinoma: a retrospective cohort study. BMJ Open 2:e000825. 10.1136/bmjopen-2012-000825 22403344 PMC3308260

[B61] Liu C. Eng C. Shen J. Lu Y. Takata Y. Mehdizadeh A.. (2016a). Serum exosomal miR-4772-3p is a predictor of tumor recurrence in stage II and III colon cancer. Oncotarget 7, 76250–76260. 10.18632/oncotarget.12841 27788488 PMC5342811

[B62] Liu L. Zheng Y.-H. Han L. Qin S.-K. (2016b). Efficacy and safety of the oxaliplatin-based chemotherapy in the treatment of advanced primary hepatocellular carcinoma: a meta-analysis of prospective studies. Medicine 95:e4993. 10.1097/MD.0000000000004993 27749557 PMC5059059

[B63] Liu Y. Liu R. Yang F. Cheng R. Chen X. Cui S. (2017). miR-19a promotes colorectal cancer proliferation and migration by targeting TIA1. Mol. Cancer 16:53 10.1186/s12943-017-0625-8 28257633 PMC5336638

[B64] Liu Z. Qiu M. Tang Q. L. Liu M. Lang N. Bi F. (2010). Establishment and biological characteristics of oxaliplatin-resistant human colon cancer cell lines. Chin. J. Cancer 29, 661–667. 10.5732/cjc.009.10666 20591218

[B65] Liu Z. Sun M. Lu K. Liu J. Zhang M. Wu W.. (2013). The long noncoding RNA HOTAIR contributes to cisplatin resistance of human lung adenocarcinoma cells via downregualtion of p21(WAF1/CIP1) expression. PLoS ONE 8:e77293. 10.1371/journal.pone.0077293 24155936 PMC3796503

[B66] Long M. Zhan M. Xu S. Yang R. Chen W. Zhang S. (2017). miR-92b-3p acts as a tumor suppressor by targeting Gabra3 in pancreatic cancer. Mol. Cancer 16:167 10.1186/s12943-017-0723-7 29078789 PMC5659029

[B67] Longley D. B. Harkin D. P. Johnston P. G. (2003). 5-fluorouracil: mechanisms of action and clinical strategies. Nat. Rev. Cancer 3, 330–338. 10.1038/nrc1074 12724731

[B68] Lu Y. Wei G. Liu L. Mo Y. Chen Q. Xu L.. (2017). Direct targeting of MAPK8IP1 by miR-10a-5p is a major mechanism for gastric cancer metastasis. Oncol. Lett. 13, 1131–1136. 10.3892/ol.2016.5544 28454224 PMC5403407

[B69] Lyskjær I. Rasmussen M. H. Andersen C. L. (2016). Putting a brake on stress signaling: miR-625-3p as a biomarker for choice of therapy in colorectal cancer. Epigenomics 8, 1449–1452. 10.2217/epi-2016-0128 27779424

[B70] Majidinia M. Yousefi B. (2016). Long non-coding RNAs in cancer drug resistance development. DNA Repair 45, 25–33. 10.1016/j.dnarep.2016.06.003 27427176

[B71] Matsumura T. Sugimachi K. Iinuma H. Takahashi Y. Kurashige J. Sawada G.. (2015). Exosomal microRNA in serum is a novel biomarker of recurrence in human colorectal cancer. Br. J. Cancer 113, 275–281. 10.1038/bjc.2015.201 26057451 PMC4506387

[B72] Mattick J. S. Makunin I. V. (2006). Non-coding RNA. Hum. Mol. Genet. 15, R17–R29. 10.1093/hmg/ddl046 16651366

[B73] Matzke M. A. Mosher R. A. (2014). RNA-directed DNA methylation: an epigenetic pathway of increasing complexity. Nat. Rev. Genet. 15, 394–408. 10.1038/nrg3794 24805120

[B74] Meister G. Tuschl T. (2004). Mechanisms of gene silencing by double-stranded RNA. Nature 431, 343–349. 10.1038/nature02873 15372041

[B75] Mercer T. R. Dinger M. E. Mattick J. S. (2009). Long non-coding RNAs: insights into functions. Nature Rev. Genet. 10, 155–159. 10.1038/nrg2521 19188922

[B76] Merhautova J. Hezova R. Poprach A. Kovarikova A. Radova L. Svoboda M. (2015). miR-155 and miR-484 are associated with time to progression in metastatic renal cell carcinoma treated with sunitinib. Biomed Res. Int. 2015:5 10.1155/2015/941980 PMC4433647 26064968

[B77] Miao Y. Zheng W. Li N. Su Z. Zhao L. Zhou H.. (2017). MicroRNA-130b targets PTEN to mediate drug resistance and proliferation of breast cancer cells via the PI3K/Akt signaling pathway. Sci. Rep. 7:41942. 10.1038/srep41942 28165066 PMC5292739

[B78] Milane L. Singh A. Mattheolabakis G. Suresh M. Amiji M. M. (2015). Exosome mediated communication within the tumor microenvironment. J. Control. Release 219, 278–294. 10.1016/j.jconrel.2015.06.029 26143224

[B79] Miyoshi J. Toden S. Yoshida K. Toiyama Y. Alberts S. R. Kusunoki M.. (2017). MiR-139-5p as a novel serum biomarker for recurrence and metastasis in colorectal cancer. Sci. Rep. 7:43393. 10.1038/srep43393 28262692 PMC5338356

[B80] Morgan R. G. (1989). Leucovorin enhancement of the effects of the fluoropyrimidines on thymidylate synthase. Cancer 63, 1008–1012. 2521810 10.1002/1097-0142(19890315)63:6+<1008::aid-cncr2820631303>3.0.co;2-z

[B81] Murtaza M. Dawson S.-J. Pogrebniak K. Rueda O. M. Provenzano E. Grant J.. (2015). Multifocal clonal evolution characterized using circulating tumour DNA in a case of metastatic breast cancer. Nat. Commun. 6:8760. 10.1038/ncomms9760 26530965 PMC4659935

[B82] Nies A. T. Magdy T. Schwab M. Zanger U. M. (2015). Role of ABC transporters in fluoropyrimidine-based chemotherapy response. Adv. Cancer Res. 125, 217–243. 10.1016/bs.acr.2014.10.007 25640272

[B83] Nohata N. Hanazawa T. Kinoshita T. Inamine A. Kikkawa N. Itesako T.. (2013). Tumour-suppressive microRNA-874 contributes to cell proliferation through targeting of histone deacetylase 1 in head and neck squamous cell carcinoma. Br. J. Cancer 108, 1648–1658. 10.1038/bjc.2013.122 23558898 PMC3668462

[B84] Noordhuis P. Holwerda U. Van Der Wilt C. L. Van Groeningen C. J. Smid K. Meijer S.. (2004). 5-Fluorouracil incorporation into RNA and DNA in relation to thymidylate synthase inhibition of human colorectal cancers. Ann. Oncol. 15, 1025–1032. 10.1093/annonc/mdh264 15205195

[B85] Olive V. Bennett M. J. Walker J. C. Ma C. Jiang I. Cordon-Cardo C.. (2009). miR-19 is a key oncogenic component of mir-17-92. Genes Dev. 23, 2839–2849. 10.1101/gad.1861409 20008935 PMC2800084

[B86] Pan J. Li X. Wu W. Xue M. Hou H. Zhai W.. (2016). Long non-coding RNA UCA1 promotes cisplatin/gemcitabine resistance through CREB modulating miR-196a-5p in bladder cancer cells. Cancer Lett. 382, 64–76. 10.1016/j.canlet.2016.08.015 27591936

[B87] Panczyk M. (2014). Pharmacogenetics research on chemotherapy resistance in colorectal cancer over the last 20 years. World J. Gastroenterol. 20, 9775–9827. 10.3748/wjg.v20.i29.9775 25110414 PMC4123365

[B88] Perez-Carbonell L. Sinicrope F. A. Alberts S. R. Oberg A. L. Balaguer F. Castells A.. (2015). MiR-320e is a novel prognostic biomarker in colorectal cancer. Br. J. Cancer 113, 83–90. 10.1038/bjc.2015.168 26035698 PMC4647533

[B89] Peters W. A Liu P. Y. Ii R. J. B. Stock R. J. Monk B. J. Alberts D. S. (2000). Concurrent chemotherapy and pelvic radiation therapy compared with pelvic radiation therapy alone as adjuvant therapy after radical surgery in high-risk early-stage cancer of the cervix. J. Clin. Oncol. 18, 1606–1613. 10.1200/JCO.2000.18.8.1606 10764420

[B90] Rasmussen M. H. Jensen N. F. Tarpgaard L. S. Qvortrup C. Rømer M. U. Stenvang J.. (2013). High expression of microRNA-625-3p is associated with poor response to first-line oxaliplatin based treatment of metastatic colorectal cancer. Mol. Oncol. 7, 637–646. 10.1016/j.molonc.2013.02.016 23506979 PMC5528477

[B91] Salzman J. (2016). Circular RNA Expression: its potential regulation and function. Trends Genet. 32, 309–316. 10.1016/j.tig.2016.03.002 27050930 PMC4948998

[B92] Shen Y. Tong M. Liang Q. Guo Y. Sun H. Q. Zheng W.. (2017). Epigenomics alternations and dynamic transcriptional changes in responses to 5-fluorouracil stimulation reveal mechanisms of acquired drug resistance of colorectal cancer cells. Pharmacogenomics J. 18, 23–28. 10.1038/tpj.2016.91 28045128 PMC5817391

[B93] Skinner H. D. Lee J. H. Bhutani M. S. Weston B. Hofstetter W. Komaki R.. (2014). A validated miRNA profile predicts response to therapy in esophageal adenocarcinoma. Cancer 120, 3635–3641. 10.1002/cncr.28911 25091571 PMC4239178

[B94] Takahashi M. Cuatrecasas M. Balaguer F. Hur K. Toiyama Y. Castells A.. (2012). The clinical significance of MiR-148a as a predictive biomarker in patients with advanced colorectal cancer. PLoS ONE 7:e46684. 10.1371/journal.pone.0046684 23056401 PMC3463512

[B95] Torre L. A. Bray F. Siegel R. L. Ferlay J. Lortet-Tieulent J. Jemal A. (2015). Global cancer statistics, 2012. CA Cancer J. Clin. 65, 87–108. 10.3322/caac.21262 25651787

[B96] Tripathi V. Shen Z. Chakraborty A. Giri S. Freier S. M. Wu X.. (2013). Long noncoding RNA MALAT1 controls cell cycle progression by regulating the expression of oncogenic transcription factor B-MYB. PLoS Genet. 9:e1003368. 10.1371/journal.pgen.1003368 23555285 PMC3605280

[B97] Ul Hussain M. (2012). Micro-RNAs (miRNAs): genomic organisation, biogenesis and mode of action. Cell Tissue Res. 349, 405–413. 10.1007/s00441-012-1438-0 22622804

[B98] US Preventive Services Task Force Bibbins-Domingo, K. Grossman D. C. Curry S. J. Davidson K. W. Epling J. W. Jr. (2016). Screening for colorectal cancer: Us preventive services task force recommendation statement. JAMA 315, 2564–2575. 10.1001/jama.2016.5989 27304597

[B99] Van Der Wilt C. L. Pinedo H. M. Smid K. Cloos J. Noordhuis P. Peters G. J. (1992). Effect of folinic acid on fluorouracil activity and expression of thymidylate synthase. Semin. Oncol. 19, 16–25. 1532671

[B100] Van Roosbroeck K. Fanini F. Setoyama T. Ivan C. Rodriguez-Aguayo C. Fuentes-Mattei E.. (2017). Combining anti-mir-155 with chemotherapy for the treatment of lung cancers. Clin. Cancer Res. 23, 2891–2904. 10.1158/1078-0432.CCR-16-1025 27903673 PMC5449263

[B101] Veettil S. K. Lim K. G. Chaiyakunapruk N. Ching S. M. Abu Hassan M. R. (2016). Colorectal cancer in Malaysia: its burden and implications for a multiethnic country. Asian J. Surg. 40, 481–489. 10.1016/j.asjsur.2016.07.005 27492344

[B102] Volpe T. Martienssen R. A. (2011). RNA interference and heterochromatin assembly. Cold Spring Harb. Perspect. Biol. 3:a003731. 10.1101/cshperspect.a003731 21441597 PMC3181039

[B103] Wang H. Li Q. Tang S. Li M. Feng A. Qin L.. (2017). The role of long noncoding RNA HOTAIR in the acquired multidrug resistance to imatinib in chronic myeloid leukemia cells. Hematology 22, 208–216. 10.1080/10245332.2016.1258152 27875938

[B104] Wang Y. Zhou B. P. (2013). Epithelial-mesenchymal transition—A hallmark of breast cancer metastasis. Cancer Hallmarks 1, 38–49. 10.1166/ch.2013.1004 24611128 PMC3944831

[B105] Wang Z. Wang Z. (2018). Downregulation of microRNA-17-5p inhibits drug resistance of gastric cancer cells partially through targeting p21. Oncol. Lett. 15, 4585–4591. 10.3892/ol.2018.7822 29541229 PMC5835921

[B106] Wilson B. J. Schatton T. Zhan Q. Gasser M. Ma J. Saab K. R.. (2011). ABCB5 identifies a therapy-refractory tumor cell population in colorectal cancer patients. Cancer Res. 71, 5307–5316. 10.1158/0008-5472.CAN-11-0221 21652540 PMC3395026

[B107] Woynarowski J. M. Faivre S. Herzig M. C. S. Arnett B. Chapman W. G. Trevino A. V.. (2000). Oxaliplatin-induced damage of cellular DNA. Mol. Pharmacol. 58, 920–927. 10.1124/mol.58.5.920 11040038

[B108] Wu C. Li M. Hu C. Duan H. (2014a). Prognostic role of microRNA polymorphisms in patients with advanced esophageal squamous cell carcinoma receiving platinum-based chemotherapy. Cancer Chemother. Pharmacol. 73, 335–341. 10.1007/s00280-013-2364-x 24288122

[B109] Wu Q. Yang Z. An Y. Hu H. Yin J. Zhang P.. (2014b). MiR-19a/b modulate the metastasis of gastric cancer cells by targeting the tumour suppressor MXD1. Cell Death Dis. 5:e1144. 10.1038/cddis.2014.110 24675462 PMC3973221

[B110] Xu K. Liang X. Cui D. Wu Y. Shi W. Liu J. (2013). miR-1915 inhibits Bcl-2 to modulate multidrug resistance by increasing drug-sensitivity in human colorectal carcinoma cells. Mol. Carcinog. 52, 70–78. 10.1002/mc.21832 22121083

[B111] Xu K. Liang X. Shen K. Sun L. Cui D. Zhao Y.. (2012). MiR-222 modulates multidrug resistance in human colorectal carcinoma by down-regulating ADAM-17. Exp. Cell Res. 318, 2168–2177. 10.1016/j.yexcr.2012.04.014 22677042

[B112] Yamamoto K. Ito S. Hanafusa H. Shimizu K. Ouchida M. (2015). Uncovering direct targets of MiR-19a involved in lung cancer progression. PLoS ONE 10:e0137887. 10.1371/journal.pone.0137887 26367773 PMC4569347

[B113] Yan B. X. Ma J. X. (2012). Promoter-associated RNAs and promoter-targeted RNAs. Cell. Mol. Life Sci. 69, 2833–2842. 10.1007/s00018-012-0953-1 22415323 PMC11114990

[B114] Yang F. Bi J. Xue X. Zheng L. Zhi K. Hua J.. (2012). Up-regulated long non-coding RNA H19 contributes to proliferation of gastric cancer cells. FEBS J. 279, 3159–3165. 10.1111/j.1742-4658.2012.08694.x 22776265

[B115] Yang Y. Jiang C. Yang Y. Guo L. Huang J. Liu X.. (2018). Silencing of LncRNA-HOTAIR decreases drug resistance of non-small cell lung cancer cells by inactivating autophagy via suppressing the phosphorylation of ULK1. Biochem. Biophys. Res. Commun. 497, 1003–1010. 10.1016/j.bbrc.2018.02.141 29470986

[B116] Ye F.-G. Song C.-G. Cao Z.-G. Xia C. Chen D.-N. Chen L.. (2015). Cytidine deaminase axis modulated by miR-484 differentially regulates cell proliferation and chemoresistance in breast cancer. Cancer Res. 75, 1504–1515. 10.1158/0008-5472.CAN-14-2341 25643696

[B117] Yee J. Weinstein S. Morgan T. Alore P. Aslam R. (2013). Advances in CT colonography for colorectal cancer screening and diagnosis. J. Cancer 4, 200–209. 10.7150/jca.5858 23459511 PMC3584833

[B118] Yu D. D. Lv M. M. Chen W. X. Zhong S. L. Zhang X. H. Chen L.. (2015). Role of miR-155 in drug resistance of breast cancer. Tumor Biol. 36, 1395–1401. 10.1007/s13277-015-3263-z 25744731

[B119] Zhang J. Xiao Z. Lai D. Sun J. He C. Chu Z.. (2012). miR-21, miR-17 and miR-19a induced by phosphatase of regenerating liver-3 promote the proliferation and metastasis of colon cancer. Br. J. Cancer 107, 352–359. 10.1038/bjc.2012.251 22677902 PMC3394980

[B120] Zhang J. Zhang K. Bi M. Jiao X. Zhang D. Dong Q. (2014). Circulating microRNA expressions in colorectal cancer as predictors of response to chemotherapy. Anticancer. Drugs 25, 346–352. 10.1097/CAD.0000000000000049 24304648

[B121] Zhang W. Lin J. Wang P. Sun J. (2017). miR-17-5p down-regulation contributes to erlotinib resistance in non-small cell lung cancer cells. J. Drug Target. 25, 125–131. 10.1080/1061186X.2016.1207647 27633093

[B122] Zhang Y. Hu X. Miao X. Zhu K. Cui S. Meng Q.. (2016). MicroRNA-425-5p regulates chemoresistance in colorectal cancer cells via regulation of programmed cell death 10. J. Cell. Mol. Med. 20, 360–369. 10.1111/jcmm.12742 26647742 PMC4727563

[B123] Zhang Y. Wang J. (2017). MicroRNAs are important regulators of drug resistance in colorectal cancer. Biol. Chem. 398, 929–938. 10.1515/hsz-2016-0308 28095367 PMC5911396

[B124] Zou J. Liu L. Wang Q. Yin F. Yang Z. Zhang W.. (2017). Downregulation of miR-429 contributes to the development of drug resistance in epithelial ovarian cancer by targeting ZEB1. Am. J. Transl. Res. 9, 1357–1368. 28386361 PMC5376026

